# Physiological signal analysis and open science using the Julia language and associated software

**DOI:** 10.3389/fnetp.2024.1478280

**Published:** 2024-11-06

**Authors:** George Datseris, Jacob S. Zelko

**Affiliations:** ^1^ Department of Mathematics and Statistics, University of Exeter, Exeter, United Kingdom; ^2^ Department of Mathematics, Northeastern University, Boston, MA, United States; ^3^ OHDSI Center, Roux Institute, Northeastern University, Portland, ME, United States

**Keywords:** digital signal processing, physiological signals, complexity measures, Julia, time series analysis, reproducible, open science

## Abstract

In this mini review, we propose the use of the Julia programming language and its software as a strong candidate for reproducible, efficient, and sustainable physiological signal analysis. First, we highlight available software and Julia communities that provide top-of-the-class algorithms for all aspects of physiological signal processing despite the language’s relatively young age. Julia can significantly accelerate both research and software development due to its high-level interactive language and high-performance code generation. It is also particularly suited for open and reproducible science. Openness is supported and welcomed because the overwhelming majority of Julia software programs are open source and developed openly on public platforms, primarily through individual contributions. Such an environment increases the likelihood that an individual not (originally) associated with a software program would still be willing to contribute their code, further promoting code sharing and reuse. On the other hand, Julia’s exceptionally strong package manager and surrounding ecosystem make it easy to create self-contained, reproducible projects that can be instantly installed and run, irrespective of processor architecture or operating system.

## 1 Introduction

Progress in modern physiological signal processing relies on research software tools that support general digital signal processing procedures and measurements within reproducible workflows. However, as the field advances, old software is susceptible to being outdated, inaccessible (due to licensing fees or expired download links), or unusable because of decaying infrastructure that renders such tools inoperable. To mitigate this issue, the field needs modern tools and infrastructure that are designed to fulfill three criteria:

1. Encourage the reuse and maintenance of existing infrastructure.
2. Easily allow software composition and extensions to accommodate new methods.
3. Be rooted in, and by design and adoption, follow open-source and open science principles.

Proprietary software typically fails to meet these criteria. For example, proprietary platforms are typically closed source (e.g., MATLAB) and do not allow users to extend or maintain software by themselves but only through paid sponsorship. In contrast, the Python programming language, which is highly popular in academia, satisfies all three of these criteria.

In this mini review, we discuss how the Julia programming language (Bezanson et al. 2017; Bezanson et al. 2012) is a strong candidate that also addresses these criteria and can be advantageous for open science in physiological signal processing. When compared with Python, Julia has better computational performance for typical user-written code (Bezanson et al., 2017), which becomes particularly important in physiological signal processing, where time series hypothesis testing is common (see Section 3). The design of Julia empirically leads to stronger software composition, similar to what is seen in Python packages (Karpinski, 2019; White, 2020). In addition to these two points, Julia also has a plethora of existing software for digital signal processing workflows (some of which are unique to the language), has excellent interoperability with other programming languages, showcases strong code reuse throughout its ecosystems, offers exceptional reproducibility infrastructure, and is based on overwhelmingly open and community-based software development practices.

## 2 Digital signal processing

### 2.1 File I/O

The start of a typical DSP analysis workflow is file I/O, which is straightforward within Julia. The FileIO ecosystem provides I/O machinery for a variety of data formats (hierarchical data, imaging, etc.) unified into one standardized interface, while the JuliaHealth organization (and associated groups) provides support for specialized physiological data formats. In Julia, most data loading operations return data in the form of a “dataframe.” First originating in R and popularized further through the tidyverse (Wickham et al., 2019) and pandas (Wes, 2010) ecosystems, this data structure in Julia is implemented through the package DataFrames.jl (Bouchet-Valat and Kamiński, 2023) and further described by the interface package Tables.jl. Because of this, several ecosystems across Julia have opted to support this interface, allowing the ready application of various software methods to signals and simplifying one’s workflow needs.

Furthermore, some particular physiological data I/O capabilities within Julia are

1. Medical Imaging Data: DICOM.jl and DICOMTree.jl for reading, writing, and viewing DICOM image data and associated metadata; NIfTI.jl for reading MRI NIfTI files, and BIDSTools.jl for working with the Brain Imaging Data Structure.
2. Time series: NeuroAnalyzer.jl (Wysokiński, 2024) supports the ability to load EEG, MEG, NIRS, MEP, and other body sensor data, and KomaMRI.jl (Castillo-Passi et al., 2023) supports loading of MRI signals and image formats.
3. Patient Medical Records: FHIRClient.jl for connecting to FHIR servers and building SMART on FHIR applications, EDF.jl for manipulating EDF/EDF+ and BDF files, and OMOPCDMCohortCreator.jl (Zelko et al., 2024) for working with OMOP CDM formatted patient databases.

Finally, Julia provides support for other data formats used broadly across different ecosystems. For example, one of the most common data formats, “delimited files” (such as CSVs and TSVs), is broadly supported by CSV.jl (Quinn et al., 2023). For data formats from proprietary tools (where the format is publicly disclosed), there is support in Julia for several tools such as XLSX.jl for Microsoft Excel, ReadStatTables.jl for STATA files, and MAT.jl for the various versions of MATLAB mat files. Additionally, for other more specialized data formats, there are tools like HDF5.jl for hierarchical data files, Arrow.jl for Apache Arrow binary files, and packages such as Tar.jl for other compressed data files. Finally, if support for a particular format is not existent or robust enough within Julia, one can use a variety of interoperability packages from JuliaInterop to supplement one’s Julia workflow with other ecosystems.

### 2.2 DSP in Julia

Building upon the compositional aspects of Julia, several tools have been created to analyze signals or time series within Julia such as

1. Traditional DSP methods: DSP.jl (Kornblith et al., 2023) is the largest package with a collection of “traditional” DSP algorithms. It includes periodogram and parametric estimation, filter design and filtering methods, window functions, convolutions, and more. AdaptiveFilters.jl provides adaptive filtering. Additionally, SignalAnalysis.jl complements and extends DSP.jl with additional functionality such as time–frequency analysis, Wigner–Wille distributions, or DEMON spectra.
2. Signal alignment and comparison: SignalAlignment.jl attempts to align signals either through shifting or warping methods (DynamicAxisWarping.jl; Bagge Carlson and Chitre (2020) provided the time warping methods used within SignalAlignment.jl). SpectralDistances.jl (Bagge Carlson and Chitre, 2020) also examines signals primarily in the frequency domain via optimal-transport distance metrics as an extension to Distances.jl.
3. Direct spectral transforms: Julia has several packages for transforming time series into spectral space: FFTW.jl and AbstractFFTs.jl for standard Fourier transforms, NFFT.jl (Knopp et al., 2023), LPVSpectral.jl (Bagge Carlson et al., 2017); ?, and FastTransforms.jl for non-equidistant transforms, and Wavelets.jl for wavelet transforms.
4. Noise reduction and signal decomposition: SignalDecomposition.jl is used for de-noising signals via decomposition, and KissSmoothing.jl is used for smoothing (other tools like convolutions and wavelet transforms can also be used directly for smoothing). For multidimensional data, there is SingularSpectrumAnalysis.jl, while principal component analysis exists within MultivariateStats.jl.
5. Hypothesis testing: HypothesisTests.jl provides a plethora of standard statistical tests. Time seriesSurrogates.jl (Haaga and Datseris, 2022) combined with ComplexityMeasures.jl (§3) provides tests for determining the nature of the system generating the signals. Associations.jl (Haaga and RomeoV, 2024) provides several methods for independence and dependence testing between signals.
6. Optimization: Julia’s flagship optimization package, JuMP (Lubin et al., 2023), along with its subpackage, Convex.jl (Udell et al., 2014, can be used to solve a variety of optimization problems that arise during DSP workflows, eliminating the need to resolve to a specialized optimization package.

In addition to these general DSP tools, specific physiological signal analysis tools also exist such as

1. Neurophysiological signal processing: NeuroAnalyzer.jl (Wysokiński, 2024) is a robust Julia toolbox for reviewing neurophysiological data. It provides several methods including loading recordings for EEG, MEG, NIRS, MEP, and other body sensors; processing methods (ICA, PCA, NIRS, etc.); analysis of specific neurological responses (ERPs, EROs, etc.); visualizations; and more.
2. MRI signal simulation: KomaMRI.jl (Castillo-Passi et al., 2023) is a Julia package for highly efficient MRI simulations. It focuses on simulating scenarios that could arise in pulse sequence development and offers several methods and comprehensive tutorials for MRI signal analysis.

### 2.3 Toolboxes and code reuse

It is common that in other programming ecosystems, physiological digital signal processing toolboxes exist. For example, for neurophysiological signal processing, there is EEGLAB (Delorme and Makeig, 2004) in MATLAB, and within Python, there is mne-python (Gramfort et al., 2013). In Julia, there exists NeuroAnalyzer.jl (Wysokiński, 2024) and KomaMRI.jl (Castillo-Passi et al., 2023). The BrainFlow toolbox (Parfenov et al., 2023) also has a Julia implementation.

However, due to the strong inter-package communication, facilitated largely by the language design and the multiple dispatch system, functionalities that exist in one package can be reused in another one. This often removes the need for creating dedicated toolboxes that bring many tools together; in the majority of cases, the tools already work together out of the box.

Additionally, due to Julia’s interoperability with other languages, one may easily utilize, for example, Python packages within Julia using PythonCall.jl. The same applies to software written in C, FORTRAN, R, MATLAB, and other programming languages. This inter-operability makes Julia conducive to code reuse and a strong candidate for sustainable software development.

## 3 Complexity measures for signal processing

Complexity measures are one of the most well-established tools in physiological time series analysis, as evidenced by hundreds of software programs created for physiological complexity analysis (Mayor et al., 2021). Complexity measures are statistics derived from time series that quantify some property of the underlying dynamics generating the time series. They have been used to distinguish determinism from noise (Rosso et al., 2007), classify time series into classes with different dynamics (Zanin and Olivares, 2021; Mayor et al., 2023), quantify directional associations between time series (Vejmelka and Paluš, 2008), and more.

Julia is the basis for the software ComplexityMeasures.jl (Datseris and Haaga, 2024). It provides thousands of complexity measure estimators out of the box, and a recent objective comparison shows it to outclass alternative software programs in terms of computational performance, reliability, total number of features, and extensibility (see Table 1 in the study by Datseris and Haaga (2024)). ComplexityMeasures.jl is a component of the DynamicalSystems.jl (Datseris, 2018) software library for nonlinear dynamics and time series analysis. ComplexityMeasures.jl integrates fully with Time seriesSurrogates.jl (Haaga and Datseris, 2022), a highly optimized and, to the best of our knowledge, the most extensive software program for surrogate hypothesis testing. The combination of ComplexityMeasures.jl with Time seriesSurrogates.jl is routinely 1,000× faster than alternatives (Datseris and Haaga, 2024). These aspects make ComplexityMeasures.jl a unique advantage of the Julia language in the context of physiological signal processing.

Perhaps even more relevant for this mini review however is that ComplexityMeasures.jl follows an open community approach in its development practices, and actively invites practitioners to become part of the software by contributing their new algorithms to it directly [see Conclusions in Datseris and Haaga (2024)]. This is particularly relevant for open and reproducible science because 1) it can decrease reproducibility issues in complexity measure applications and 2) merge the currently disparate efforts on software for complexity measures; so far, hundreds of such software programs have been created, often with minimal differences between them, essentially putting more effort into replication than into new progress.

Hence, by design, ComplexityMeasures.jl plays an instrumental role in promoting open science in physiological signal processing. By being well-documented, inviting practitioners with its open development practices, and being exceptionally well-tested, it provides a guarantee on maintaining high-quality and open-source implementations of existing algorithms and enabling trustworthy and reproducible physiological signal processing. For more details, refer to Datseris and Haaga (2024) and the list of software programs in the supplementary material provided by Mayor et al. (2021) for further comparison.

The usage of ComplexityMeasures.jl is straightforward. A single function called complexity (or entropy, depending on the measure) may estimate the measure by taking as an input 1) the measure to estimate and 2) the input time series (univariate, multivariate, or spatiotemporal). As such, ComplexityMeasures.jl is designed to be composable not only with the whole Julia ecosystem but also with any programming environment due to its simple interface. The code example in Section 6 shows its application to EEG data.

## 4 Julia efficiency for numerical computing

Another aspect of the Julia language that affects time series analysis and beyond is the efficiency of getting work done with Julia itself. One can quickly prototype algorithm implementations or analysis pipelines within Julia due to its simple and high-level syntax. Sometimes, even these prototype implementations within Julia can meet the performance needs of individuals due to its robustly constructed just-in-time (JIT) compiler. Then, by optimizing one’s code within Julia itself through various approaches, highly performant code can be created that is competitive at the level of languages that are commonly regarded as high performance (i.e., C, Fortran, or Rust) (Godoy et al., 2023), without the need for language extensions or re-writing code in another language.

## 5 Open and reproducible science with Julia

### 5.1 Vibrant open-source community

One of the most crucial aspects of the Julia programming language is not a technical contribution but rather the Julia community itself. There are several official community platforms where Julia discussions and collaborations take place, totaling tens of thousands of active participants and hundreds of thousands of messages being shared. Additionally, as an emergent property, the overwhelming majority of projects in the Julia programming language are hosted publicly on GitHub, an international platform fostering open-source code collaboration.

Within the Julia community, self-organized communities have organically emerged to specifically leverage Julia for common tasks across several domains. In particular, many tools for domains of work and research are centered within these Julia “organizations,” such as

1. JuliaHealth: an organization that leverages Julia to improve health and medical research. They organize monthly meetings with organization members, and anyone interested in getting started in research software development, conducting novel health research, or sharing a question or work they have done can join. Moreover, they centralize some smaller workgroups focused on areas such as medical imaging, standards and interoperability, and more within health research contexts.
2. JuliaInterop: while Julia is still growing, JuliaInterop exists to bridge packages and workflows from other languages into Julia. As a result, Julian are free to use their favorite tools from other languages within Julia workflows while also maintaining a presence within these other language communities.
3. JuliaML: utilizing Julia’s priority support for numerical methods, JuliaML gathers for monthly community meetings to discuss the latest developments in machine learning research. In these meetings, development discussions are common to triage what various members are developing and what they need within the JuliaML ecosystem for their work.
4. JuliaDynamics: an umbrella organization for Julia software related to nonlinear dynamics, nonlinear time series analysis, and complex systems. They organize monthly meetings showcasing interesting applications of the software in real-world problems and discussing future developments for the organization and its software.

These “organizations” are not incorporated in any official sense but serve as gathering points for interested practitioners and volunteers to coalesce tools they have been developing and share expert insights. To the best of our knowledge, other languages generally do not have as strong social coherence. For example, in Python, we could not find such self-organized organizations for DSP or Health, perhaps because functionality tends to be aggregated into huge infrastructures like NumPy, making it daunting for individuals to become involved. In contrast, within the Julia community, this self-organization is much more common and robust; practically, every (sub) field of science has an associated Julia GitHub organization that is self-organized and not owned by a corporation or large research group. This may be facilitated by characteristics of Julia, such as multiple dispatch and the subsequent package communication it provides, allowing small projects to grow while still being part of a greater whole. These aspects of Julia result in a low contribution barrier: a researcher can easily turn their scripts into published source code within a registered Julia package.

### 5.2 Package manager

One of the biggest strengths of the Julia language is its “package manager,” Pkg.jl, which is a Julia software program that installs “packages” (individual Julia software). Hence, installing new packages happens from within the language itself and allows full access to all of Julia’s infrastructure during installation. Pkg.jl is the only package manager in Julia, and it defines and records dependencies in only one way.

A critical feature of Pkg.jl is its support for “environments.” Julia environments are self-contained Julia projects that have their own list of dependencies and installed packages. This allows one to use different versions of the same software package across Julia environments. The latest version of one package can be used within new projects, while older project environments can safely continue to use older versions in old projects, greatly alleviating dependency hell problems (Wikipedia contributors, 2024).

Each Pkg.jl environment is governed by two configuration files: Project.toml and Manifest.toml. The Project.toml is a user-created file that declares the direct dependencies of the project, optionally declaring compatibility bounds. The Manifest.toml is a Julia-generated file that lists the current full dependency tree of the environment and is updated each time any package update is done in the environment. The Manifest.toml file can be sent to another user, who can then instantiate an identical environment (with respect to the package versions) as the original created environment.

Pkg.jl also does exceptionally well in a problem that older programming languages still struggle with: robust installation. It provides the “artifact system,” provisioned via Yggdrasil.jl and BinaryBuilder.jl. An “artifact” within Julia is any raw data file or precompiled binary dependency (i.e., a piece of software program that exists from outside of Julia). Developers may distribute these artifacts during the installation of their package by utilizing this system. As a result, installing an “artifact” from scratch is not a problem that every single user of one’s Julia software has to solve; rather, it is a problem that needs to be solved once by a software developer through configuring the needed artifacts. The user then “simply installs” their Julia package, and the artifacts are shipped to them during the installation. Additionally, the artifacts are versioned in the same way as normal packages and hence are also included in the aforementioned Manifest.toml file, further enabling reproducibility.

### 5.3 Other projects fostering sharing and reproducibility

In addition to Pkg.jl, Julia has a couple of highly popular projects to further facilitate scientific reproducibility. Pluto.jl is a programming notebook alternative to Jupyter (Kluyver et al., 2016) that places a strong focus on the reproducibility and accessibility of the code. It solves some of the reproducibility problems related to Jupyter, such as hidden variables or translating a notebook to pure source code, and it also provides a reactive environment for accelerating code development and/or scientific workflows.

DrWatson (Datseris et al., 2020) is scientific project assistant software. It simplifies and accelerates managing a scientific software project by setting up simulations or processing workflows. Like Pluto.jl, it places a strong focus on accessibility and reproducibility and provides several functionalities for making a scientific project more robust and easier to share and reproduce. DrWatson also has the benefit of being completely non-invasive in contrast to many other similar software programs [see comparison provided by Datseris et al. (2020)]. DrWatson is used like a typical Julia package: a user may use any of its exported functions in their source code or scripts without altering any of the surrounding code.

## 6 Example application

**FIGURE 1:**
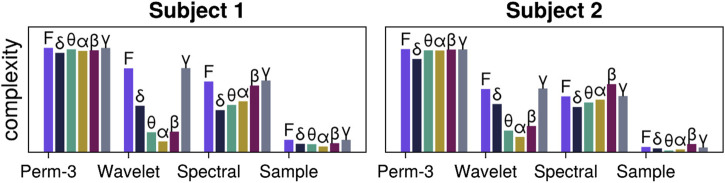
Exemplary time series analysis of EEG signals from one channel. EEG signals for two subjects are first decomposed into frequency bands (with “F” standing for the Full EEG signal that has not been decomposed and only been passed through a denoising filter). For each signal and band, we estimate various complexity measures, in particular order-3 permutation entropy (Bandt and Pompe, 2002), wavelet entropy (Rosso et al., 2001), spectral entropy (Tian et al., 2017), and sample entropy (Richman and Moorman, 2000). See Listing 1 for the code that produced the figure.

As a simple application, we showcase a code snippet that performs two actions: first, it decomposes an input EEG time series into time series containing frequencies from the characteristic frequency bands: δ,θ,α,β,γ , using NeuroAnalyzer.jl (Wysokiński, 2024). This is done for two input EEG time series from two different subjects obtained from *A Resting-state EEG Dataset for Sleep Deprivation* (Xiang et al., 2024). Then, for each time series and some of the frequency bands, we estimate various complexity measures via ComplexityMeasure.jl (Datseris and Haaga, 2024). The result is presented as a barplot in Figure 1, and the code that produced it is provided in Listing 1.
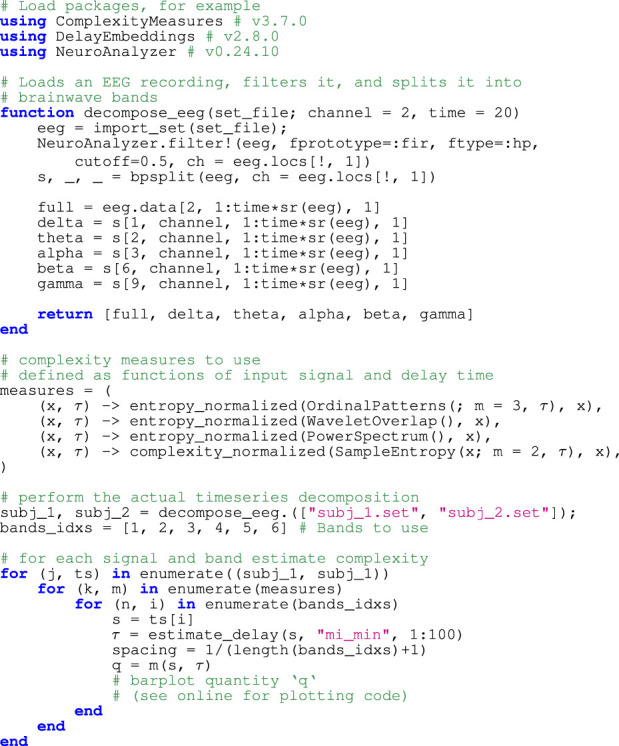

Listing 1 Example code listing for Figure 1.

See the online reproducible codebase (Datseris and Zelko, 2024) associated with this paper for the code that loads the time series, as well as the full dependency tree for the packages used to produce the final figure.

## 7 Conclusion

We believe that the adoption of the Julia programming language can significantly increase accessibility and reproducibility in physiological signal processing while promoting a sustainable ecosystem based on collaboration and code reuse. Julia can accelerate the development of new methods and scientific progress, in general, due to its flexible syntax, available libraries, high performance, and package interoperability. Two similar discussions to our paper, arriving at similar conclusions regarding the positives of using Julia, were recently conducted in the context of high-energy physics by Eschle et al. (2023) and biology by Roesch et al. (2023).
